# Hydrus microstent implantation for surgical management of glaucoma: a review of design, efficacy and safety

**DOI:** 10.1186/s40662-019-0157-y

**Published:** 2019-10-22

**Authors:** Saba Samet, Jeb A. Ong, Iqbal Ike K. Ahmed

**Affiliations:** 1 Department of Ophthalmology and Vision Sciences, University of Toronto, Toronto, ON Canada; 2 Prism Eye Institute, 2201 Bristol Circle, Suite 100, Oakville, ON L6H 0J8 Canada

**Keywords:** Hydrus, Schlemm’s canal, Minimally invasive glaucoma surgery, Drainage device, Glaucoma

## Abstract

With the advent of microinvasive glaucoma surgery (MIGS), the spectrum of modalities available to manage patients with this chronic and heterogeneous condition has broadened. Despite its novelty however, there has been a rapid evolution in the development of a multitude of devices, each targeting a structure along the aqueous drainage pathway. A growing body of evidence has demonstrated IOP and medication burden reduction, and a more favorable safety profile with MIGS procedures in contrast to traditional incisional surgeries. Among the array of MIGS, the Hydrus® Microstent (Ivantis, Inc., Irvine, CA) is a recent FDA approved device, designed to bypass the trabecular meshwork and provide a scaffold for Schlemm’s canal. The objective of this article is to review the Hydrus from conception to clinical use, and present data on its efficacy and safety to date. The available literature has shown promise, however inherent to all novel devices, only long-term monitoring will ensure sustained IOP control and an acceptable safety profile. Surgical advancements in glaucoma have revolutionized the field, and continued research and development will establish these approaches in clinical treatment algorithms.

## Background

Glaucoma is a leading cause of permanent blindness worldwide [1]. It is a progressive disease, which causes irreversible damage to the optic nerve and nerve fiber layer resulting in progressive visual field loss. Glaucoma has many risk factors including age, race and family history of the disease, but the only readily modifiable risk factor proven to slow the progression of visual field loss is intraocular pressure (IOP) [2–5]. There are various treatment modalities to reduce IOP including topical medications, laser treatment, microinvasive glaucoma surgeries (MIGS) and incisional surgeries.

Topical hypotensive medications are used as first line treatment for glaucoma and ocular hypertension. Medications, although efficacious, are plagued by high rates of patient non-adherence [6–8]. They are also associated with the development of dry eye and ocular surface disease [9–11]. Laser trabeculoplasty has been shown to be a safe and cost-effective method for lowering IOP [12, 13]. Most side-effects, such as conjunctival hyperemia and anterior chamber inflammation, are transient; however, there have been cases with intractable IOP elevations post procedure [14, 15]. When laser and medications fail to control IOP, traditional filtering surgery is considered. Trabeculectomy and tube shunt surgeries are very successful at IOP reduction, however they are reserved for advanced cases due to significant risks of sight-threatening complications and failure requiring reoperation [16]. In the Primary Tube Versus Trabeculectomy (PTVT) Study, complications were reported in 41 and 29% of the patients in the trabeculectomy and tube shunt groups, respectively [16].

Recently, developments in biomaterials and micro-fabrication technology have enabled the development of MIGS devices. Despite rapid evolution in the field and production of a variety of devices, several unifying features encompass the spectrum of MIGS including: ab interno micro-incisional approach, minimal anatomical alterations, effective IOP reduction, and improved safety profile and post-operative recovery [17]. Thus, MIGS fill the void left by previous treatment algorithms for refractive glaucoma not yet warranting the risk of traditional incisional surgery. MIGS devices lower IOP by three mechanisms: i) bypassing trabecular outflow [18]; ii) increasing uveoscleral/suprachoroidal/supraciliary outflow [19]; and iii) increasing subconjunctival outflow [20]. The Hydrus® Microstent (Ivantis, Inc., Irvine, CA) is part of the MIGS category of devices which bypass trabecular outflow. Published results suggest that the Hydrus is safe and efficacious for the treatment of open-angle glaucoma. The Hydrus device received the European CE mark of approval in 2011 and recently received FDA approval in 2018 for use in combination with phacoemulsification based on results from the 24-month HORIZON Trial [21]. The objective of this article is to review the design, efficacy and safety of the Hydrus Microstent.

## Main text

### Device and procedure

**Fig. 1:**
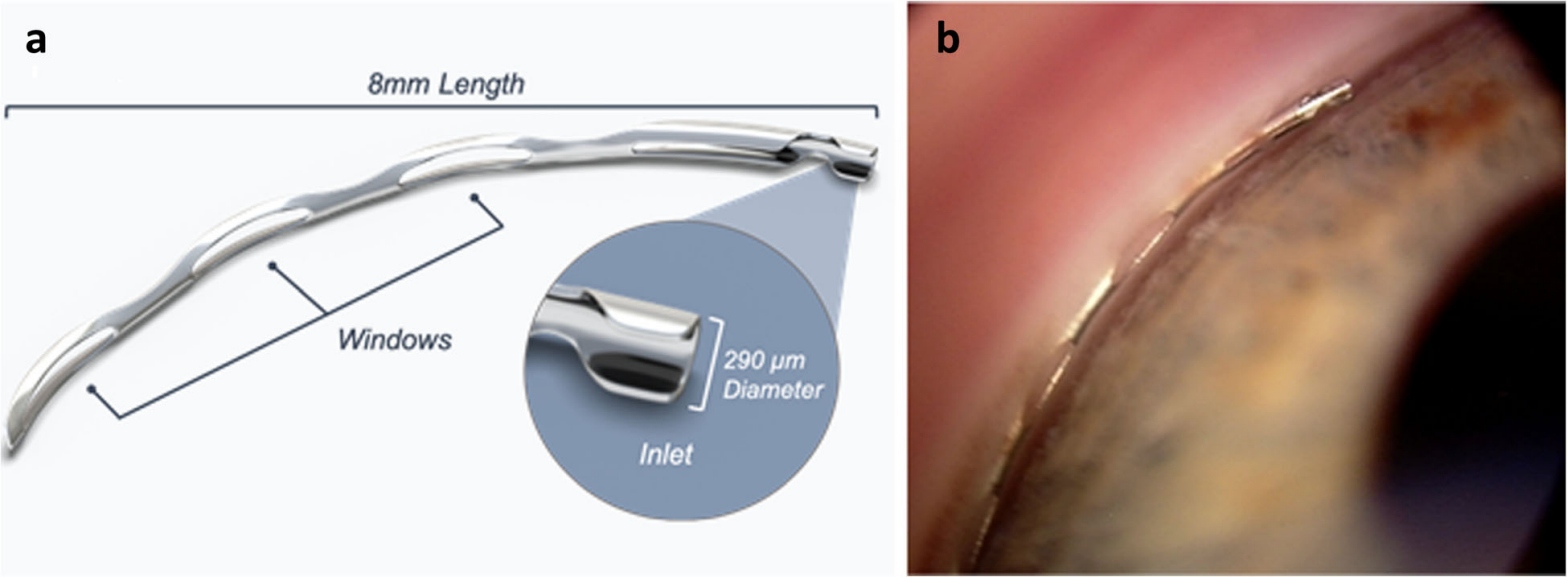
Schematic (**a**) and gonioscopic image (**b**) of the Hydrus microstent

The Hydrus is a flexible aqueous drainage device designed to be placed ab-interno where it bypasses the trabecular meshwork (TM) and dilates approximately three clock hours of Schelmm’s canal (SC). The inlet remains in the anterior chamber (AC) while the remainder of the device is placed into SC (Fig. 1). The Hydrus design thus serves to provide an alternate route to aqueous humor that otherwise faces resistance at the juxtacanalicular segment of the TM and SC inner wall, and further provides an intracanalicular scaffold for SC, providing a route for outflow to multiple collector channels [22].

Implantation of the Hydrus is performed via a peripheral clear corneal incision using a preloaded hand-held injector. Subsequent or prior to routine phacoemulsification, the microscope and patient head are adjusted to allow for a clear view of the nasal angle structures using a surgical gonioprism. Ophthalmic viscosurgical device is introduced to further fill the AC and expand the angle. The microstent is entered into the AC through the clear corneal incision and the TM is incised with the tip of the cannula. The microstent is then advanced to span approximately 90 degrees of SC, while the 1–2 mm inlet segment is left to reside in the AC. Once appropriate device positioning is confirmed, the device injector is withdrawn and viscoelastic removed.

### Ex vivo studies

#### Design and biocompatibility

The microstent has an 8 mm flexible, non-luminal open structure with windows and spines. The inlet provides a maximal SC dilation four to five times the normal SC cross-sectional area, occupying 90 degrees of SC along the scaffold length [23]. It has been reported that SC collapses with increasing IOP as a result of bowing of the TM and SC inner wall toward the outer SC wall [24], with possible herniation of TM tissue into collector channel ostia at higher pressures [25]. The Hydrus scaffold therefore provides the theoretical benefit of maintaining the SC lumen over its course, for collector channel accessibility.

The device structure is made from nitinol (55% nickel - 45% titanium alloy) and thermally set during the production process to correspond with the SC curvature. Nitinol has had applications in medical devices since the 1970s [26]. As a result of its superelasticity, biocompatibility, shape memory [27–29], as well as its non-mutagenic and non-cytotoxic properties [30, 31], it has been utilized in a variety of locations including the cardiovascular system, tendon, bladder, and the middle ear to name a few [32–36]. The ocular application of nitinol has been reported in a subretinal drug delivery system [37]. Preclinical studies of anterior chamber nitinol clips on the iris surface have further demonstrated intraocular biocompatibility [38].

**Fig. 2:**
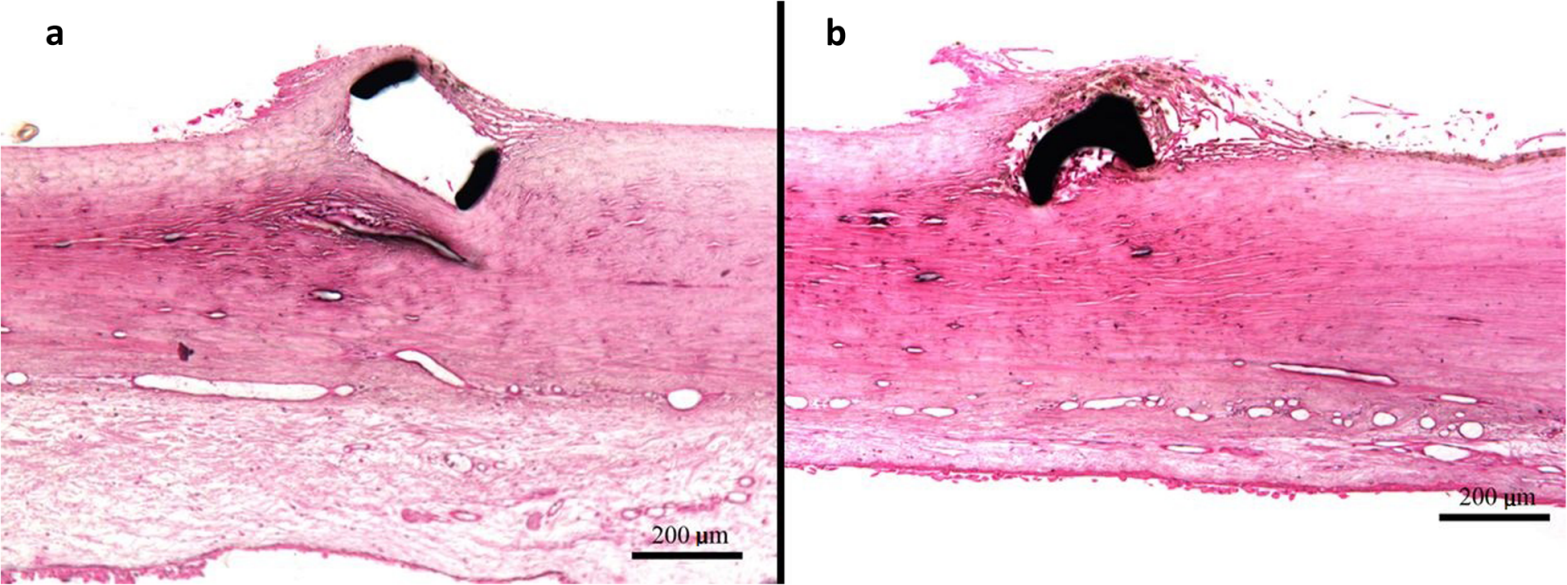
Hydrus and iStent devices in situ. (**a**) Histological section of the Hydrus scaffold window region in situ showing SC dilatation. (**b**) Histological section of the iStent micro-bypass rail in situ. Images courtesy of Hays et al. [41]

To specifically investigate the impact of Hydrus implantation on ocular tissue, several studies have carried out histological analyses post ex vivo insertion. In one of the earliest studies by Camras et al. [39] using the initial 15 mm microstent scaffold design, at the completion of outflow facility assessments, one pair of enucleated human eyes was histologically examined for microstent placement and TM appearance. Cross-sections of regions of the eyes with the Hydrus showed dilated SC, with visibly intact and similarly stretched TM. Microscopic examination to identify breaks in the SC was not performed, however as outflow facility reduced with removal of the Hydrus, if breaks are a mechanism for increasing outflow facility as proposed in the case of canaloplasty, they had little effect in this study [39, 40]. Similarly, in a subsequent study by Hays et al. [41] comparing the 8 mm Hydrus to two iStent Trabecular Micro-Bypass devices (Glaukos Inc., San Clemente CA), one human anterior segment containing a scaffold and one containing two iStents were histologically analyzed. Both the Hydrus and the iStent were reported to dilate SC and stretch the TM without breaks or discontinuity to the TM, however the microstent had more distinct SC lumen and dilatation (Fig. 2), and the extrascleral tissue with the Hydrus was wider than the tissue with the iStent [41]. The authors attributed this finding to the higher volume of fluid that flowed from collector channels into the sclera and conjunctiva with the Hydrus scaffold.

**Fig. 3:**
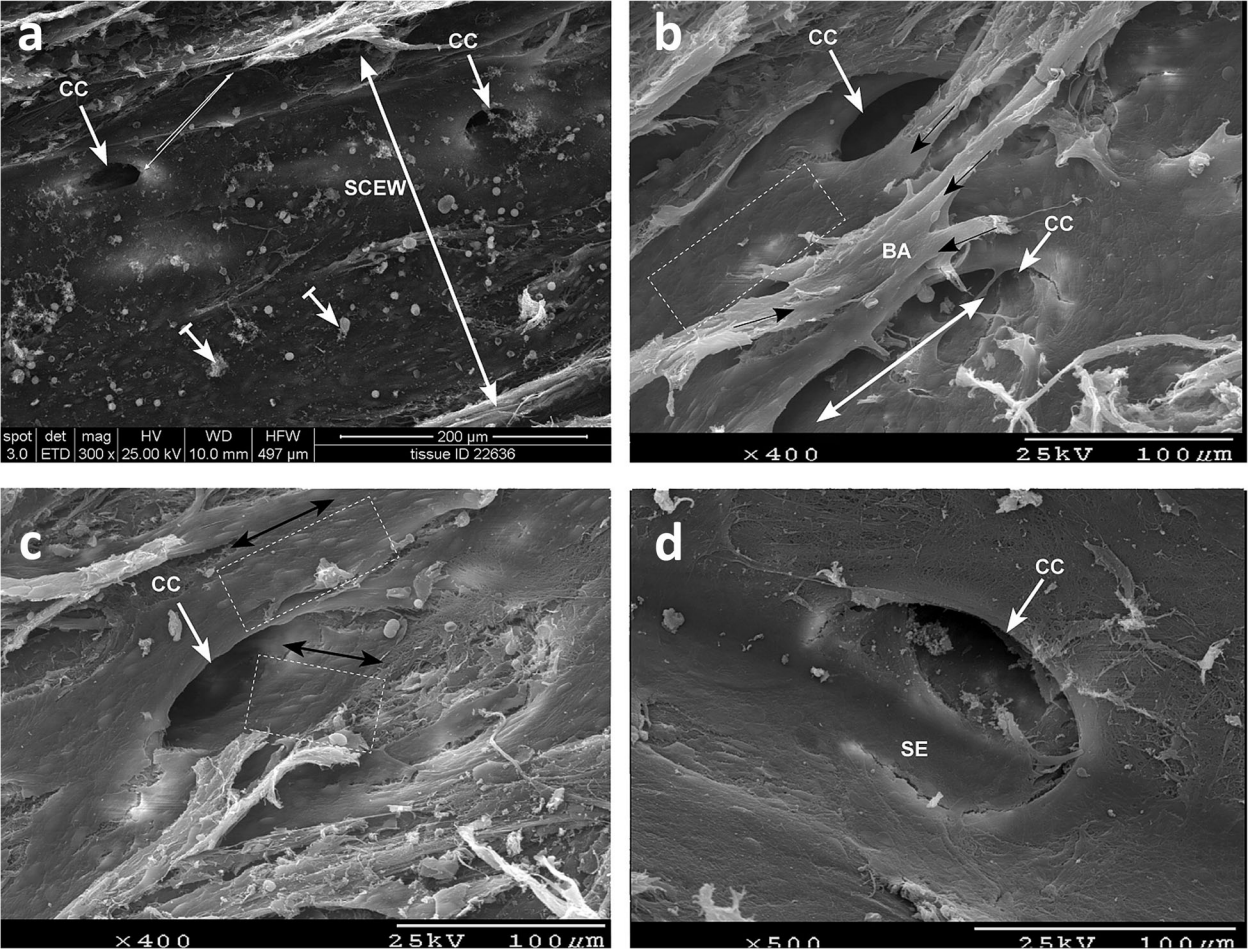
Scanning electron microscopic image of SC outer wall following insertion and removal of an 8 mm Hydrus microstent, with collector channel ostia shown in panels **a**-**d**. Particulate debris visible in image (**a**) (barred arrows). The intact but sloping edge of the collector channel ostium (shown in **d**) resulting from microstent-dependent indentation appearing to compress the lower portion of the ostia while leaving the upper portion open. Courtesy of Johnstone et al. [22]

An ex vivo study of three human anterior segments implanted with the 8 mm microstent, two with the 15 mm microstent and six controls was conducted by Johnstone et al. [22], to assess the distribution of irregular particulate matter (IPM), shape of collector channel (CC) ostia, and health of the SC endothelium using scanning electron microscopy (SEM). The CCs did not show evidence of obstruction, compression, or margin disruption, and particulate debris did not appear to occlude SC (Fig. 3). In areas of microstent contact for both the 8 mm and 15 mm scaffolds, CCs were patent and intact with indentations free of particulate debris, however the SC external wall showed a smaller area of indentation with the 8 mm microstent [22]. The study demonstrated minimal disruption to SC and CC anatomy and patency, with the 8 mm design having a lower potential for CC obstruction due to reduced contact with SC outer wall.

The Hydrus microstent has further demonstrated biocompatibility in adult New Zealand white rabbit and cynomolgus non-human primate (NHP) models [42]. At the time of manufacturing, biocompatibility was enhanced by electropolishing the microstent to passivate the surface and replace corrosive metallic elements with a non-reactive titanium oxide layer. Subsequent testing verified corrosion resistance of the surface [43, 44], and SEM inspection demonstrated smooth surface and edges [42]. Two NHP eyes received Hydrus implantation and one eye received sham surgery as a control. In vivo clinical examinations and IOP measures were within normal limits during the 13-week post-implantation follow-up period. Post euthanasia, encapsulation was assessed using the Jansen qualitative and semi-quantitative grading scheme, and light microscopy and SEM used to inspect for debris, fibrin formation and tissue damage. In the area of the microstent, loss of TM tissue volume varied from partial loss to loss of recognizable features of the TM and SC likely secondary to tissue compression. Except for a few mononuclear cells and a thin capsule wall scored as Jansen 4, the physiological response was minimal with no evidence of inflammation, granuloma formation, or metallosis. Similarly, in the adult rabbit arm, one of each pair of eyes received the Hydrus with the contralateral eye receiving sham procedure. In vivo examinations demonstrated low-grade anterior uveitis and hyphema with all cases resolved by the first postoperative month. Subsequent to euthanasia at 26-weeks, light microscopy revealed minimal mononuclear cell infiltration and fibrotic response, with stent encapsulation of Jansen 3–4 grading. This was despite implantation of the microstent in highly vascularized and reactive orbital, extraocular muscle and conjunctival tissue in several cases.

The initial series of histological studies confirm minimal immediate mechanical effects of SC instrumentation, yet there are shortcomings with regard to the absence of bleeding, inflammatory and scarring processes in ex vivo models that may alter histopathology. The study by Grierson et al. [42], however, supports the biocompatibility of the nitinol scaffold implant for intraocular implantation, suggesting that appropriate clinical evaluations can be conducted.

#### Efficacy – outflow facility and resistance

The initial set of preclinical studies additionally investigated the efficacy of the 8 mm and 15 mm Hydrus scaffolds using outflow facility and resistance measures. Each of the three ex vivo experiments by Camras et al. [39], Gulati et al. [23], and Hays et al. [41] were conducted using human anterior segment models connected to a perfusion pressure system, with outflow facility then measured and averaged at perfusions of 10, 20, 30, and 40 mmHg (with the addition of 50 mmHg in the case of Hays et al.’s [41] Hydrus versus 2 iStent study) at baseline and post implantation/sham procedure. All data are presented as mean ± SD unless otherwise noted.

Table 1 summarizes the results of outflow facility and resistance experiments. For the 15 mm scaffold design, nine anterior segments received the Hydrus and seven had a sham procedure. Mean outflow facility increased from 0.19 ± 0.02 to 0.39 ± 0.07 μL/min/mmHg (mean ± SEM, *n* = 9, *p* < 0.01) with the Hydrus, and 0.20 ± 0.03 to 0.23 ± 0.03 μL/min/mmHg (mean ± SEM, *n* = 7, *p* > 0.05) in controls. With removal of the Hydrus, outflow facility subsequently returned to baseline values. The log-converted ratio of post implantation to baseline outflow facility was significantly higher in experimental eyes (2.11 ± 0.312, mean ± SEM) than in controls (1.27 ± 0.16, mean ± SEM) at all perfusion pressure levels (*p* < 0.05) except for 10 mmHg due to high outflow facility variability. Furthermore, outflow facility increased more with perfusion pressure increases in eyes implanted with the Hydrus compared to baseline (*p* < 0.05, *n* = 9), whereas controls did not show a significant difference in this relationship from baseline to post-sham procedure. This initial study on the 15 mm scaffold therefore illustrated that the Hydrus increases outflow facility independent of the implantation procedure, with a greater improvement seen at higher pressures.

**Table 1:** Summary of outflow facility and resistance studies

Publication	Intervention	N Anterior Segments	Baseline Outflow Facility (μL/min/mmHg)	Outflow Facility Post-Implant (μL/min/mmHg)	*p*	Baseline Outflow Resistance (mmHg/μL/min)	Outflow Resistance Post-Implant (mmHg/μL/min)	*p*
Camras et al. 2012 [39]	Hydrus 15 mm	9	0.19 ± 0.02	0.39 ± 0.07	< 0.01	–	–	–
	Controls	7	0.20 ± 0.03	0.23 ± 0.03	> 0.05	–	–	–
Gulati et al. 2013 [23]	Hydrus 8 mm	24	0.33 ± 0.17	0.52 ± 0.19	< 0.001	4.38 ± 3.03	2.34 ± 1.04	< 0.001
	Controls	24	0.39 ± 0.21	0.38 ± 0.19	0.82	4.30 ± 3.64	3.47 ± 1.68	0.31
Hays et al. 2014 [41]	Hydrus 8 mm	12	0.28 ± 0.10	0.44 ± 0.13	0.001	4.30 ± 1.91	2.68 ± 1.16	0.0016
	2 iStents	12	0.29 ± 0.09	0.37 ± 0.12	0.046	4.05 ± 1.42	3.17 ± 1.18	0.004

The 8 mm scaffold, open configuration design was investigated in 24 Hydrus implanted eyes and 24 contralateral eye controls. Outflow facility increased from 0.33 ± 0.17 to 0.52 ± 0.19 μL/min/mmHg (mean ± SD, *n* = 24, *p* < 0.001) in experimental eyes, and 0.39 ± 0.21 to 0.38 ± 0.19 μL/min/mmHg (*n* = 24, *p* = 0.014) in controls. Similarly, outflow resistance decreased from 4.38 ± 3.03 to 2.34 ± 1.04 mmHg/μL/min (*p* < 0.001) in experimental eyes, and 4.30 ± 3.64 to 3.47 ± 1.68 mmHg/μL/min (*p* = 0.31) in controls. Outflow facility was found to increase with a corresponding decrease in resistance at all levels of perfusion pressure. Gulati et al. [23] found a linear correlation between baseline outflow resistance and resistance reduction (*R*^*2*^ = 0.89, *p* < 0.0001). As such, the study confirms that success with outflow facility improvement extends to the newer 8 mm Hydrus design with IOP lowering effect potentially higher with higher baseline outflow resistance or preoperative IOP.

Owing to the difference in baseline outflow facility values, direct comparisons between the 8 mm and 15 mm designs are difficult to make. Gulati et al. [23] calculated standardized mean difference between pre and post microstent insertion means using Hedge’s unbiased *g* for the two studies. Although they found a higher effect size for outflow facility with the 15 mm scaffold (*g* = 1.23 versus *g* = 0.98), this can be attributed to lower baseline outflow facility in the 15 mm microstent study [23]. They found no significant difference in trend lines between baseline outflow resistance and change in outflow resistance between the two studies. This analysis suggests no theoretical dissimilarity in efficacy between the 8 mm open and 15 mm long, circular Hydrus designs. Although the 15 mm scaffold has the ability to extend to more CCs, the area of indentation and potential for CC obstruction with SC outer wall contact seems to offset this benefit [22].

One study has directly compared the 8 mm Hydrus with 2 iStent implants in 12 pairs of eyes [41]. Mean outflow facility increased from 0.28 ± 0.10 to 0.44 ± 0.13 μL/min/mmHg (0.16 ± 0.12 μL/min/mmHg increase, *n* = 12, *p* = 0.001) with Hydrus insertion, and 0.29 ± 0.09 to 0.37 ± 0.12 μL/min/mmHg (0.08 ± 0.12 μL/min/mmHg increase, *n* = 12, *p* = 0.046) with iStent insertion. The Hydrus scaffold resulted in a significantly greater mean outflow facility improvement (*p* = 0.03), as well as individual outflow facility increase at perfusion pressures of 30, 40, and 50 mmHg (*p* < 0.05) compared to the iStent. Furthermore, the Hydrus resulted in an outflow resistance reduction of 4.30 ± 1.91 to 2.68 ± 1.16 mmHg/μL/min (1.62 ± 1.35 mmHg/μL/min decrease, *p* = 0.0016), while the iStent reduced resistance from 4.05 ± 1.42 to 3.17 ± 1.18 (0.89 ± 0.85 mmHg/μL/min decrease, *p* = 0.004), with a significantly greater reduction by the Hydrus (*p* = 0.035). Hays et al. [41] also confirmed previous findings of the association between higher baseline resistance and greater post implantation resistance reduction (*R*^*2*^ = 0.68, *p* = 0.002), with no significant correlation found in iStent cases (*R*^*2*^ = 0.31, *p* = 0.06).

The aforementioned set of preclinical investigations lend support to the efficacy of the Hydrus device. It is important, however, to mention limitations that are common to all stated studies. Ex vivo models lack scarring and inflammatory physiological responses which may hinder expected IOP reduction results. Furthermore, these simulations lack episcleral venous pressure as well as uveoscleral outflow pathways, which are important variables in aqueous humor dynamics for determination of final IOP. Nevertheless, the investigations have developed a foundation sufficient for shifting device evaluations to the surgical setting.

### Clinical studies

Table 2 outlines a summary of studies evaluating the Hydrus microstent (HM).

**Table 2:** Efficacy results of Hydrus Microstent studies

Publication	Study Design	N Eyes at Baseline	N Eyes at Follow-up	Glaucoma Type	Follow-up (months)	Baseline IOP Medicated (M) Washed-Out (W)	IOP (mmHg) at Follow-up (months)	Baseline Meds	Meds at Follow-up (Months)	Success / Failure Criteria	% Successful at (Months)	Reoperation Rate
Gandolfi et al. 2016 [45]	Retrospective comparative case series	21 Hydrus	100%	12 POAG 7 PXG 2 PDG	24	24.0 ± 6.0 (M)	15.0 ± 3.0 (24)	3.1 ± 0.6	0.9 ± 0.9 (24)	To achieve post-surgery ‘target’ IOP (mid-high teens): Complete success = no meds Qualified success = some meds Failure = glaucoma surgery	Complete = 33.3% (24) Qualified = 57.1% (24) Failure = 9.5% (24)	9.5%
		24 Canaloplasty	100%	16 POAG 8 PXG		26.0 ± 4.0 (M)	16.0 ± 2.0 (24)	2.7 ± 0.8	0.7 ± 0.9 (24)		Complete = 50.0% (24) Qualified = 41.7% (24) Failure = 8.3% (24)	8.3%
Fea et al. 2017 [46]	Retrospective case series	92 Hydrus + Phaco	80% (12 m) 73% (24 m)	84 POAG 7 PXG 1 PDG	24	19.4 ± 4.4 (M)	15.5 ± 2.7 (12) 15.7 ± 2.5 (24)	2.1 ± 1.0	0.6 ± 1.0 (12) 0.7 ± 1.0 (24)	1) unmedicated IOP ≤18 2) unmedicated IOP ≤15	1) 70% (12) 1) 52% (24) 2) 36% (12) 2) 25% (24)	0% (12 m) 1% (24 m)
Fea et al. 2017 [51]	Prospective interventional comparative case series	31 Hydrus	97%	POAG	12	23.1 ± 5.1 (M)	16.5 ± 2.6 (12)	2.29 ± 0.83	0.9 ± 1.04 (12)	Target IOP (mid-teens) maintained with no medication	47% (12)	0%
		25 SLT	100%	POAG		23.2 ± 2.2 (M)	15.9 ± 2.5 (12)	2.48 ± 0.92	2.0 ± 0.91 (12)		4% (12)	0%
Al-Mugheiry et al. 2017 [52]	Prospective observational cohort	25 Hydrus + Phaco	100% (12 m)	21 POAG 2 NTG 2 PXG	16.8 ± 5.6 (12–24)	18.1 ± 3.6 (M)	15.3 ± 2.2 (Last f/u)	1.96 ± 0.96	0.04 ± 0.20 (Last f/u)	1) unmedicated IOP < 21 2) unmedicated IOP < 18 3) unmedicated IOP < 15	1) 96% (Last f/u) 2) 80% (Last f/u) 3) 32% (Last f/u)	0%
Pfeiffer et al. 2015 [53]	Randomized Controlled Trial, single masked, multicentre	50 Hydrus + Phaco	96% (12 m) 96% (24 m) 92% W (12 m) 88% W (24 m)	45 POAG 5 PXG	24	18.9 ± 3.3 (M) 26.3 ± 4.4 (W)	16–17 (12) (M) 16–17 (24) (M) 16.6 ± 2.8 (12) (W) 16.9 ± 3.3 (24) (W)	2.0 ± 1.0	0.5 ± NR (12) 0.5 ± 1.0 (24)	≥20% reduction in washed out diurnal IOP	88% (12) 80% (24)	0% (12 m) 2.1% (24 m)
		50 Phaco	98% (12 m) 90% (24 m) 88% W (12 m) 68% W (24 m)	41 POAG 8 PXG 1 PDG		18.6 ± 3.8 (M) 26.6 ± 4.2 (W)	16–17 (12) (M) 16–17 (24) (M) 17.4 ± 3.7 (12) (W) 19.2 ± 4.7 (24) (W)	2.0 ± 1.1	NR ± NR (12) 1.0 ± 1.0 (24)		74% (12) 46% (24)	0% (12 m) 4.1% (24 m)
Samuelson et al. 2019 [21]	Randomized Controlled Trial, single masked, multicentre	369 Hydrus + Phaco	95%	POAG	24	17.9 ± 3.1 (M) 25.5 ± 3.0 (W)	16.8 ± 3.2 (24) (M) 17.4 ± 3.7 (24) (W)	1.7 ± 0.9	0.3 ± 0.8 (24)	≥20% reduction in washed out diurnal IOP	85.9% (12) 77.3% (24)	0%
		187 Phaco		POAG		18.1 ± 3.1 (M) 25.4 ± 2.9 (W)	17.4 ± 3.0 (24) (M) 19.2 ± 3.8 (24) (W)	1.7 ± 0.9	0.7 ± 0.9 (24)		70.0% (12) 57.8% (24)	2.1%
Ahmed et al. 2019 [56]	Randomized Controlled Trial, single masked, multicentre	75 Hydrus	97% 40% W	72 POAG 3 PXG/PDG	12	19.0 ± 3.9 (M) 27.5 ± 4.4 (W)	17.3 ± 3.7 (24) (M) 21.5 ± NR (24) (W)	2.5 ± 0.7	1.0 ± NR (24)	IOP ≤18, no meds, no secondary glaucoma surgery/ trabeculoplasty/cataract surgery	35.6% (12)	0
		77, 2 iStents	97% 31% W	71 POAG 6 PXG/PDG		19.1 ± 3.6 (M) 27.3 ± 4.2 (W)	18.1 ± 3.7 (24) (M) 23.3 ± NR (24) (W)	2.7 ± 0.8	1.7 ± NR (24)		10.5% (12)	2.6%

*IOP* intraocular pressure, *M* medicated, *W* washed-out, *POAG* primary open angle glaucoma, *PXG* pseudoexfoliation glaucoma, *PDG* pigmentary glaucoma, *NTG* normal tension glaucoma, *SLT* selective laser trabeculoplasty, *NR* not reported, *Last f/u* last follow up, *m* month

#### Retrospective series

Gandolfi et al. [45] compared 21 cases of standalone HM to 24 cases of ab-externo canaloplasty (CP) in a retrospective comparative case series including patients with primary or secondary open-angle glaucoma and 24 months follow-up. All canaloplasty procedures were completed using the iTrack 250A microcatheter (iScience Interventional, Inc., Menlo Park, CA). Both groups had similar baseline characteristics with regard to demographics, IOP, hypotensive medications, and previous treatment with argon laser trabeculoplasty/selective laser trabeculoplasty (ALT/SLT). The mean medicated baseline IOP in the HM group was 24.0 ± 6.0 mmHg decreasing significantly to 15.0 ± 3.0 mmHg at post-operative month 24 (*p* = 0.001). The CP group had a similar IOP trend decreasing from 26.0 ± 4.0 to 16.0 ± 2.0 mmHg (*p* = 0.001), with no statistically significant difference between the two groups (*p* = 0.18). The number of baseline medications was not reported numerically, however can be calculated as 3.1 ± 0.6 and 2.7 ± 0.8 (Fig. 2 of Gandolfi et al. [45]) with reduction to 0.9 ± 0.9 and 0.7 ± 0.9 at 24 months in the HM and CP groups, respectively. There was no significant difference in the medication regimen intensity (i.e., number of patients on 0, 1 or more active substances) between the two groups (*p* = 0.74). Complete success was defined as achieving “target” post-operative IOP (mid-high teens) on 0 medications at the 24-month time point; 33.3% of HM and 50.0% of CP patients met this standard. 57.1% of HM and 41.7% of CP participants were deemed qualified successes as they attained “target” IOP with medication. Two patients from each group were counted as failures due to requiring additional glaucoma surgery. The distribution of clinical success and failures between the two groups were not significantly different. This study also looked at the effect of previous laser trabeculoplasty on complete success. Previous ALT/SLT resulted in a lower complete success rate in the CP group compared to the HM group (*p* = 0.04), although further studies will need to be conducted to confirm this result. Among the few studies assessing visual fields, Hydrus-implanted patients had a reduction in visual field mean defect from 4.6 ± 1.9 to 4.2 ± 1.9, with CP patients having a reduction in mean defect from 4.0 ± 3.2 to 3.9 ± 3.3 by 2 years, with no significant intergroup difference detected at either time point. With regard to intraoperative complications, none were reported. Transient post-operative hyphema was the most common complication at 19.0% in the HM group and 29.2% in the CP group. YAG laser for lysis of peripheral anterior synechiae (PAS) was required in 4 cases of the HM arm of the study. Table 3 summarizes all safety results.

**Table 3:** Safety results of Hydrus Microstent studies

Publication	Intervention	Hyphema	Corneal Pathology	Focal PAS/ Iris Adhesions	Device Obstruction	Device Malposition	Laser for PAS/ Iris Adhesion	IOP Spike	Secondary Surgical Intervention	Other
Gandolfi et al. 2016 [45]	21 Hydrus	19.0%	–	–	–	–	19.0%	4.8%	9.5%	–
	24 Canaloplasty	29.2%	–	–	–	–	25.0%	12.5%	8.3%	–
Fea et al. 2017 [46]	92 Hydrus + Phaco	1.1%	–	8.7%	1.1%	1.1%	1.1%	–	1.1% Trab	CME 1.1% CRVO 1.1%
Fea et al. 2017 [51]	31 Hydrus	6.4%	3.2% Edema	–	–	–	–	6.4%	–	**–**
	25 SLT	–	–	–	–	–	–	–	–	Mild eye discomfort 40%
Al-Mugheiry et al. 2017 [52]	25 Hydrus + Phaco	36.0%	28.0% Edema	20.0%	4.0%	–	–	4.0% POD1, 20.0% POW1	–	Anterior uveitis 48.0% POD1, 28.0% POW1 CME 4.0% Stent-iris touch 4.0% PCO 16.0%
Pfeiffer et al. 2015 [53]	50 Hydrus + Phaco	–	2.0% POM1 Descemet folds	12.0% POY1 18.8% POY2	–	–	–	4.0% POY1	2.1% POY2	Mac edema 2.0% POY1 Optic disc hem 2.0% POY1 Vitreal mac traction 2.1% POY2
	50 Phaco	–	2.0% POM1 Descemet folds	2.0% POY1 2.0% POY2	–	–	–	4.0% POY1	4.1% POY2	Mac edema 4.0% POY1 Vitreal mac traction 2.0% POY1 ERM 4.0% POY1, 2.0% POY2 Retinal detachment 2.0% POY1 Wound dehiscence 2.0% POY1 AION 2.0% POY1 Iris erosion 6.0% POM1
Samuelson et al. 2019 [21]	369 Hydrus + Phaco	0.5%	1.4% Edema 1.1% Abrasion	14.9%	3.8%		0.8%	0.5%	0.3% Paracentesis 0.8% Laser membranectomy	Uveitis/iritis 5.6% Conjunctivitis 5.75% CME 2.2% ERM 1.6% Subconj hem 2.4%
	187 Phaco	0.5%	–	2.1%	–	–	–	2.7%	2.1% Tube shunt/Trab 1.0% Paracentesis 0.5% SLT	Uveitis/iritis 3.7% Conjunctivitis 7.0% CME 2.1% ERM 1.6% Neovascular glaucoma 0.5%
Ahmed et al. 2019 [56]	75 Hydrus	–	–	–	12.2%	–	1.3%	4.1%	–	New cataract 2.6%
	77, 2 iStent	–	–	–	13.2%	–	–	5.2%	2.6% Trab/GDD	New cataract 1.3% Cataract surgery 1.3%

*PAS* peripheral anterior synechiae, *IOP* intraocular pressure, *CME* cystoid macular edema, *CRVO* central retinal vein occlusion, *SLT* selective laser trabeculoplasty, *PCO* posterior capsule opacification, *POD1* postoperative day 1, *POW1* postoperative week 1, *POM1* postoperative month 1, *POY1* postoperative year 1, *POY2* postoperative year 2, *ERM* epiretinal membrane, *AION* anterior ischemic optic neuropathy, *mac* macular, *subconj* subconjunctival, *hem* hemorrhage, *Trab* trabeculectomy, *GDD* glaucoma drainage device

Fea et al. [46] conducted a retrospective case series of 92 eyes with primary or secondary open-angle glaucoma receiving the Hydrus implant and phacoemulsification. Mean IOP reduced from 19.4 ± 4.4 to 15.5 ± 2.7 at 1 year and 15.7 ± 2.5 mmHg at 2 years (*p* < 0.001), with greater reduction of IOP correlating with baseline IOP (*R*^*2*^ = 0.72). The authors conducted a subgroup analysis looking at patients with baseline IOP 18 mmHg or less (Group 1, *n* = 42) and those with IOP 19 mmHg or higher (Group 2, *n* = 50). Group 1 did not have an appreciable reduction in IOP (15.8 ± 1.9 to 15.1 ± Not Reported (NR) at 1 year and 15.7 ± NR mmHg at 2 years) but did have a significant reduction in medication number (1.86 ± 0.9 to 0.2 ± 0.5 at 1 year and 0.5 ± 0.7 at 2 years, *p* < 0.0001), while Group 2 had a significant 31% reduction in IOP (22.6 ± 3.4 to 16.0 ± 3.2 at 1 year and 15.7 ± 2.3 mmHg at 2 years, *p* < 0.0001) with a less prominent but still significant reduction in medication number (2.4 ± 1.1 to 0.7 ± 1.2 at 1 year and 1.0 ± 1.2 at 2 years, *p* < 0.05). Thus, it was demonstrated that the Hydrus benefits the cohort of patients with lower preoperative IOP by reducing medication burden and maintaining IOP, while reducing both IOP and medications in patients with higher preoperative IOP. The magnitude of postoperative IOP reduction is dependent on preoperative IOP, which is similar to that found in application of the SLT, iStent, and Trabectome [47–49], and is consistent with previous ex vivo outflow facility studies. Efficacy of the microstent extended to those with severe glaucoma and previous incisional surgery as well, where 6 patients in this category had an IOP reduction of 20.2 ± 3.8 to 15.0 ± 3.0 mmHg and maintenance of medication number from 2.7 ± 0.8 to 2.5 ± 1.0 at 2 years. Success criteria of unmedicated IOP ≤18 mmHg was met by 70 and 52% of patients, and unmedicated IOP ≤15 mmHg was achieved by 36 and 25% of patients at 1 and 2 years, respectively. Intraoperative stent repositioning was required in 2 of 92 cases. The most common postoperative complication was focal iris adhesions, with 8 unobstructive cases and 1 requiring argon laser due to device obstruction.

To evaluate implant safety, Fea et al. [50] conducted a nonrandomized, retrospective study on 62 consecutive patients divided into a group affected by age-related cataract (Group 1, *n* = 25), and a group affected by cataract and primary open-angle glaucoma (Group 2A, *n* = 19 cataract surgery alone; Group 2B, *n* = 18 cataract surgery and Hydrus insertion). Using the Konan Cell Check XL (Konan Medical, Irvine, CA, USA), they found no significant differences among the groups with regard to preoperative endothelial parameters. All groups had significant change in endothelial cell density pre- and postoperatively (9.1% in Group 1, 17.24% in Group 2A and 11.71% in Group 2B), although the change in endothelium parameters with Hydrus implantation was comparable to those who underwent cataract surgery alone.

#### Prospective series

A prospective interventional comparative case series was published by Fea et al. [51] comparing 31 eyes with uncontrolled mild to moderate primary open-angle glaucoma receiving the Hydrus and 25 eyes receiving SLT (360 degrees, 100 non-overlapping spots). Target IOPs were set prior to either procedure with postoperative medication added for IOPs greater than 21 mmHg, or above the pre-set target on 3 occasions. There was no significant difference between the groups at baseline with regard to age, visual acuity, IOP, medication number, angle width and lens status, however preoperative visual field mean defect was worse in the Hydrus group (− 8.43 ± 6.84 versus − 3.04 ± 0.65). Although the SLT group experienced a greater IOP reduction in the early postoperative period (6.0 ± 3.3 versus 4.3 ± 6.8 mmHg, *p* = 0.26), there was no intergroup difference by 1 year of follow-up (6.6 ± 5.6 versus 7.3 ± 2.5 mmHg reduction in the Hydrus and SLT groups, respectively, *p* = 0.57). Medication number, however, reduced significantly by 1.4 ± 0.97 (*p* < 0.05) in the Hydrus group but only by 0.5 ± 1.05 (*p* > 0.05) in the SLT group, with a significantly higher reduction in medication burden using the Hydrus (*p* = 0.001). These results remained consistent despite adjustment using a propensity score accounting for baseline characteristics. With this analysis the authors found no significant difference in IOP at 1 year, but a higher medication number (1.19 medications more/patient) in the SLT group. By the final 1-year follow-up point, 47% of Hydrus patients were medication-free in contrast to 4% of SLT patients.

To assess the impact of learning on hypotensive effect, adverse effects, and surgical procedure duration, Al-Mugheiry et al. [52] conducted an observational cohort study of the first 25 Hydrus implantations with concomitant phacoemulsification of a single surgeon. They found no significant learning effect on outcomes; however, surgical time reduced with consecutive case number (from 30 min to < 20 min, *r* = − 0.65; *p* = 0.0005). Although results were not reported at a set time point (rather at final follow-up, mean 16.8 ± 5.6 months), they found an IOP reduction of 18.1 ± 3.6 to 15.3 ± 2.2 mmHg. Medication number of 1.96 ± 0.96 decreased to 0.04 ± 0.20 (*p* < 0.0001). Success criteria of unmedicated IOP less than 21, 18 and 15 were met by 96, 80, and 32% of patients by final follow-up. Intraoperative complications were minimal, including 2 cases of hyphema and 1 case requiring two insertion attempts.

#### Randomized controlled trials

The HYDRUS II [53] randomized controlled trial compared 50 patients receiving HM in combination with phacoemulsification with 50 receiving phacoemulsification alone in patients with primary and secondary open-angle glaucoma. Diurnal IOPs (dIOP) were obtained and medication was restarted if IOP was > 19 mmHg or with visual field/optic nerve progression. Mean washed out dIOP (WO-dIOP) at baseline was 26.3 ± 4.4 mmHg in the combined group, which declined significantly to 16.6 ± 2.8 mmHg at 1 year and 16.9 ± 3.3 mmHg at 2 years, and from 26.6 ± 4.2 mmHg in the control group to 17.4 ± 3.7 mmHg and 19.2 ± 4.7 mmHg in the cataract group at 12 and 24 months, respectively. The difference in WO-dIOP between groups at the 24-month time point (but not at 12 months) was statistically significant (*p* = 0.009). 88% of patients at 12 months and 80% at 24 months met the primary end point of 20% drop in WO-dIOP in the HM group, compared to 74 and 46% of patients in the cataract group at 12 and 24 months, respectively (not statistically different at 12 months; *p* = 0.0008 at 24 months). Baseline number of medications were compared to medications at 24 months decreasing from 2.0 ± 1.0 to 0.5 ± 1.0 in the combined group and from 2.0 ± 1.1 to 1.0 ± 1.0 in the phacoemulsification group. The difference in number of medications between groups at 24 months was statistically significant (*p* = 0.019). 72.9% of HM patients were medication free at 24 months compared to 37.8% of patients having phacoemulsification alone (*p* = 0.0008). The study was limited to 44 patients in the HM group and 34 patients in the control group who underwent washout due to exit from the study, further glaucoma surgery, safety concerns, death, and health or non-health related reasons. Focal PAS in the area of the microstent was the most frequent adverse event with 9 cases at 24 months compared to 1 in the control group (*p* = 0.008), although IOP and medication use was similar between those with PAS and the overall Hydrus group. Other complications were not significantly different between the two groups.

A comparison can be made to the Samuelson et al. [54] and Craven et al. [55] iStent with concomitant phacoemulsification versus phacoemulsification alone, randomized controlled trials. Evaluating only unmedicated subjects not requiring a postoperative washout, the between group difference (of MIGS with phacoemulsification versus phacoemulsification alone) for subjects with 20% IOP reduction at 1 year was 23% in the HYDRUS II versus 18% with the iStent. By 2 years, this was 39% with the Hydrus and 9% in the iStent. This potentially indicates that there is a more stable and long-lasting treatment effect with the Hydrus device [53].

In the HORIZON [21] clinical trial, 556 eyes with mild to moderate primary open-angle glaucoma were randomized in a 2:1 ratio to Hydrus and phacoemulsification (369), and phacoemulsification alone (187). Similar to the HYDRUS II study, mean WO-dIOP decreased from 25.5 ± 3.0 to 17.4 ± 3.7 mmHg by 24 months (7.6 ± 4.1 mmHg reduction) in the study group, and from 25.4 ± 2.9 to 19.2 ± 3.8 (5.3 ± 3.9 mmHg reduction) in the control arm. Patients with the Hydrus had a 2.3 mmHg greater WO-dIOP reduction at 24 months (*p* < 0.001, 95% CI 1.6–3.0). 85.9% of patients at 12 months and 77.3% at 24 months met the primary endpoint of 20% drop in WO-dIOP in the HM group, compared to 70.0 and 57.8% of patients in the cataract group at 12 and 24 months, respectively (*p* < 0.001 at 12 and 24 months). With a covariate analysis accounting for baseline characteristics, the response to treatment in the Hydrus group remained significantly higher than controls. The HORIZON trial also had similar medication reduction results to the HYDRUS II study, where the study arm had 1.4 reduction in medications compared to 1.0 in the control arm on average (*p* < 0.001). 78% of HM patients were medication free at 24 months versus 48% of patients having phacoemulsification alone (*p* < 0.001). Intraoperatively, there were 4 cases of hyphema, 1 cyclodialysis cleft, 1 iridodialysis, 1 malposition in the iris root and 1 Descemet membrane detachment in the Hydrus group. Focal PAS was again the most common postoperative complication at 14.9% with no significant difference in IOP reduction in patients with or without obstructive PAS. Samuelson et al. [21] also analyzed visual field data, where 4.3% of HM patients and 5.3% of controls had a worsening of mean defect by 2.5 dB at 2 years. Further studies however are required to support this finding. Cup to disk ratio and central corneal thickness however, remained stable during follow-up.

A recent paper by Ahmed et al. [56] on the COMPARE study, has evaluated the Hydrus scaffold versus 2 iStent insertions over a period of 12 months. This was a randomized controlled trial of 75 mild to moderate open-angle glaucoma patients receiving the Hydrus versus 77 receiving 2 iStent implants. All Hydrus patients had successful implantation in contrast to 97.4% success with the iStent, where in two cases, 1 iStent was inserted. The washout requirement was eliminated during the study due to concerns with iStent patients having persistent elevated IOP despite medical therapy. In the HM group, mean medicated IOP decreased from 19.0 ± 3.9 to 17.3 ± 3.7 mmHg (1.7 mmHg reduction, *p* = 0.009), while the iStent group had a decrease from 19.1 ± 3.6 to 18.1 ± 3.7 mmHg (1.0 mmHg reduction, *p* = 0.09). They found no significant between-group difference in IOP reduction (*p* = 0.3), however the Hydrus had a significantly lower percentage of patients with IOP > 21 mmHg and a significantly higher percentage of patients with IOP < 21/18/15 mmHg from preoperative to postoperative timepoints; this was not found in the iStent group. In the group of patients where washout was done, WO-dIOP reduced by 6.0 ± 5.4 mmHg (*n* = 30) and 4.0 ± 5.6 mmHg (*n* = 24) in the Hydrus and iStent groups, respectively. Consistent with previous Hydrus studies, medication number reduced by 1.6 ± 1.2 (*p* < 0.001) in HM patients and 1.0 ± 1.2 (*p* < 0.001) in iStent patients, with the Hydrus resulting in greater medication reduction (*p* = 0.004). 22.6% more patients were medication free in the Hydrus group (*p* = 0.006). 30.1% of HM patients had an unmedicated IOP ≤18 mmHg at 12 months compared to 9.3% of iStent patients (*p* = 0.002), and 39.7% of unmedicated HM patients had a 20% or more reduction in IOP from baseline washout compared to 13.3% with the iStent (*p* < 0.001). The 1-year cumulative event free survival rate (see Table 2. for definition) was 35.6% for the Hydrus and 10.5% for the iStent (*p* = 0.001). As such, the Hydrus resulted in greater complete success with less medication compared to the iStent and a similar safety profile (Table 3).

## Conclusions

The presented collection of studies from ex vivo preclinical experiments to randomized clinical trials support the surgical utility of the Hydrus MIGS device. Despite the limitations present in all studies including loss to follow-up, unmasked investigators, and potentially increased medication compliance post-procedure, the Hydrus seems to reproducibly lower IOP to the mid-high teens and reduce medication burden. The long-term efficacy of the Hydrus as well as further studies comparing MIGS devices will need to be evaluated to strongly establish the positioning of the Hydrus, and microinvasive surgeries in general, along the spectrum of glaucoma management.

## Data Availability

Data sharing is not applicable to this article as no datasets were generated or analyzed during the current study.

## Abbreviations

AC: Anterior chamber
ALT: Argon laser trabeculoplasty
CC: Collector channel
CP: Canaloplasty
dIOP: Diurnal intraocular pressure
HM: Hydrus microstent
IOP: Intraocular pressure
IPM: Irregular particulate matter
MIGS: Microinvasive glaucoma surgery
NHP: Non-human primate
NR: Not reported
PAS: Peripheral anterior synechiae
SC: Schlemm’s canal
SEM: Scanning electron microscopy
SLT: Selective laser trabeculoplasty
TM: Trabecular meshwork
TVT: Tube versus trabeculectomy
WO-dIOP: Washed out diurnal intraocular pressure
